# The Crosstalk of Epigenetics and Metabolism in Herpesvirus Infection

**DOI:** 10.3390/v12121377

**Published:** 2020-12-01

**Authors:** Yonggang Pei, Erle S. Robertson

**Affiliations:** Departments of Otorhinolaryngology-Head and Neck Surgery, and Microbiology, and the Tumor Virology Program, Abramson Cancer Center, Perelman School of Medicine at the University of Pennsylvania, Philadelphia, PA 19104, USA; peiy@pennmedicine.upenn.edu

**Keywords:** epigenetics, metabolism, herpesvirus infection

## Abstract

Epigenetics is a versatile player in manipulating viral infection and a potential therapeutic target for the treatment of viral-induced diseases. Both epigenetics and metabolism are crucial in establishing a highly specific transcriptional network, which may promote or suppress virus infection. Human herpesvirus infection can induce a broad range of human malignancies and is largely dependent on the status of cellular epigenetics as well as its related metabolism. However, the crosstalk between epigenetics and metabolism during herpesvirus infection has not been fully explored. Here, we describe how epigenetic regulation of cellular metabolism affects herpesvirus infection and induces viral diseases. This further highlights the importance of epigenetics and metabolism during viral infection and provides novel insights into the development of targeted therapies.

## 1. Introduction

Human herpesvirus family is a group of important DNA viruses that are associated with a spectrum of human diseases [1]. This family contains eight members that belong to three subgroups according to the cells they predominantly infect and the site of latency [1]. They are α-herpesvirus: herpes simplex virus 1 (HSV-1), herpes simplex virus 2 (HSV-2) and varicella zoster virus (VZV); β-herpesvirus: human cytomegalovirus (HCMV), human herpesvirus 6 (HHV-6) and human herpesvirus 7 (HHV-7); γ-herpesvirus: Epstein–Barr virus (EBV) and Kaposi’s sarcoma-associated herpesvirus (KSHV). A crucial characteristic of herpesvirus infections is their ability to switch between latency and lytic reactivation. These human herpesviruses primarily establish latency in different cell types, in particular, α-herpesviruses in sensory neurons, β-herpesviruses in mononuclear cells and γ-herpesviruses in B-cells [1,2]. They can maintain persistent latency in the host for many years to avoid immune system recognition, and they can reactivate periodically when conditions related to immune impairment are suitable, leading to asymptomatic shedding or clinical diseases.

Epigenetics, a crucial factor that affects herpesvirus infection, is characterized by DNA methylation and histone modifications. These epigenetic modifications control chromosome organization and transcriptional regulation and to a large extent determine success of infection [3]. For instance, the initial stage of HSV-1 infection is dependent on the dynamic regulation of viral chromatin structure, which restricts or activates viral gene expression with the assistance of cellular transcription factors and cofactors [4]. The status of viral genomes was regulated by enrichment of activated H3K4 methylation and H3K9 acetylation, or repressed H3K9 trimethylation and H3K27 trimethylation [2,5]. The critical roles of epigenetics in herpesvirus infection have been highlighted for some time, but the interaction between epigenetics and metabolism still needs exploration.

Metabolism includes anabolism and catabolism in cells or tissues, and its deregulation which involves dysfunction of metabolic enzymes is an important hallmark of cancer. Metabolic substrates or enzymes are key players in regulating cellular epigenetic levels via controlling DNA methylation and histone methylation or acetylation. Recent studies demonstrated the critical relationship between epigenetic reprogramming and metabolic regulation in multiple cancers, which showed the importance of targeting the key molecules or pathways as strategies for new anticancer treatment [6,7]. The crosstalk of epigenetics and metabolism directly modulates viral infection or viral-associated diseases; however, their interactions in herpesvirus research have not attracted as much attention. Our review highlights the critical roles of herpesviruses in subverting the epigenetic program and inducing diseases by regulating cellular metabolism, which includes aerobic glycolysis, fatty acid synthesis and glutaminolysis, as well as several critical metabolites, namely S-adenosylmethionine (SAM), α-ketoglutarate (α-KG), flavin adenine dinucleotide (FAD), acetyl-coenzyme A (acetyl-CoA) and nicotinamide adenine dinucleotide (NAD^+^). Understanding the interactions of epigenetics and metabolism in herpesvirus infection could provide new insights for the development of novel therapeutic strategies against herpesvirus-associated diseases.

## 2. Aerobic Glycolysis or Warburg Effect

The Warburg effect as a cancer hallmark is characterized by increased glucose consumption and decreased oxidative phosphorylation [8,9]. Glucose is normally metabolized through the tricarboxylic acid (TCA) cycle to generate ATP as cellular energy. However, cancer cells metabolize glucose through glycolysis and produce large amounts of lactate at a faster rate [10,11]. Aerobic glycolysis is widely utilized by cancer cells to generate sufficient cellular energy supporting rapid cell growth, which could be hijacked in herpesvirus-infected cells and induction of the related diseases. Recent studies have shown that herpesviruses can regulate metabolism by modulating the activity of epigenetic enzymes.

EBV latent membrane protein LMP1 induces glucose metabolism in NPC cells [12,13]. LMP1 can also enhance DNMT1 activity and regulate its mitochondrial localization. This leads to epigenetic silencing of PTEN and hypermethylation of the mitochondrial DNA (mtDNA) D-loop region [12]. In addition, LMP1 promotes glucose metabolism through stabilization of c-Myc expression and upregulation of the glycolytic hexokinase 2 (HK2) in NPC cells [13]. In EBV-infected B-cells, LMP1-activated poly(ADP-ribose) polymerase 1 (PARP1) interacts with HIF-1α as a PARylated complex and can induce aerobic glycolysis through modulation of HIF-1α downstream targets [14].

Different cellular metabolites were monitored with liquid chromatography–tandem mass spectrometry in HCMV-infected human fibroblasts. The results showed that HCMV infection markedly regulated multiple metabolic pathways, including glycolysis and pyrimidine biosynthesis [15]. The activated pyrimidine biosynthesis drives UDP-sugar biosynthesis to support HCMV infection, and inhibition of pyrimidine biosynthesis could attenuate HCMV replication [16]. An early study indicated that increased glycolysis uptake could occur in the first 20 hours after HCMV infection and was dependent on the expression of viral early genes [17]. HCMV induced expression of glucose transporter 4 (GLUT4) and hijacked GLUT4 for increased glucose uptake, which facilitates viral production through the synthesis of fatty acids [18,19]. Furthermore, HCMV infection induced the activity of AMP-activated protein kinase (AMPK), and AMPK could enhance GLUT4 transcription through phosphorylation of histone deacetylase 5 (HDAC5), a transcriptional repressor [20,21]. Besides, HCMV-induced glycolysis is mediated by calmodulin-dependent kinase kinase (CaMKK) for viral replication and production [22]. HSV-associated glycolysis activation is not dependent on CaMKK [22].

The KSHV-induced Warburg effect in latently infected endothelial cells is defined by activated aerobic glycolysis, lactic acid production and decreased oxygen consumption [23]. The Warburg effect is essential for maintenance of KSHV latency, and its inhibition could specifically induce apoptosis of KSHV-infected endothelial cells, suggesting a strategy whereby targeting glycolysis may have potential therapeutic value [23,24]. A comparative RNA-Seq analysis showed that KSHV induced glucose uptake and lactate release in hypoxia, which may be related to the DNA methyltransferases DNMT3A and DNMT3B [25]. Moreover, KSHV miRNAs induced metabolic transformation from mitochondria biogenesis to aerobic glycolysis for latency maintenance in primary dermal microvascular lymphatic endothelial cells (LECs) through suppression of the prolyl hydroxylase EGLN2 and the heat shock protein HSPA9 [26].

## 3. Fatty Acid Synthesis

Fatty acid synthesis is the formation of fatty acid that is catalyzed from acetyl-CoA, malonyl-CoA and NADPH by fatty acid synthase (FASN) [27,28]. The metabolic intermediate citrate from the TCA cycle is utilized to generate acetyl-CoA, while malonyl-CoA is catalyzed by acetyl-CoA carboxylases (ACCs) [29,30,31]. The acetyl-CoA and malonyl-CoA molecules are synthesized to palmitate as the critical product of fatty acid synthesis by FASN, and palmitate can then further produce many other fatty acids with various lengths and saturation [28,32]. Fatty acid synthesis is an important source of cellular energy and contributes to the rapidly growing cancer cells. The regulation of fatty acid synthesis is largely related to cell growth and survival, so it is no surprise that fatty acid metabolism is increasingly recognized as an essential cancer hallmark and a promising therapeutic target. Importantly, herpesviruses also modulate fatty acid synthesis during their infection. One study explored the metabolic profiles of two herpesviruses, using mass spectrometry to assess HCMV-infected fibroblast cells and HSV-1-infected epithelial cells. The results demonstrated that HCMV profoundly promoted fatty acid biosynthesis, while HSV-1 increased pyrimidine biosynthesis [33]. These findings indicated that these two herpesviruses modulated distinct and specific metabolic programs, which have the potential to be developed as targeted therapeutic therapy [19].

EBV immediate-early (IE) protein BRLF1 can induce the expression of the fatty acid synthase, which is required for expression of EBV lytic genes [34]. This induction was mediated by p38 kinase activity [34]. Further experiments showed that FASN could activate BRLF1-mediated BZLF1 transcription through removal of the suppressive epigenetic modifications [34]. Moreover, multiplexed proteomics identified EBV activated mitochondrial one-carbon (1C) metabolism pathway that drives production of nucleotide, mitochondrial NADPH and glutathione during primary EBV infection [35]. In particular, EBV latent antigen EBNA2 targeted MYC and mediated the upregulation of MTHFD2, a crucial mitochondrial 1C enzyme, which further induced metabolic regulation [35]. Therefore, the supply of NADPH regulated by EBV could affect fatty acid synthesis in EBV-infected cells.

Global metabolic profiling demonstrated that many metabolic pathways were altered in KSHV-infected endothelial cells, including glycolysis, amino acid metabolism and lipogenesis [36]. The increased synthesis of fatty acids was required for the survival of KSHV-infected endothelial cells, while inhibition of the associated signaling pathways resulted in cell apoptosis during KSHV latent infection [24,36]. The metabolic profiles are significantly different when comparing primary human B-cells to KSHV-associated primary effusion lymphoma (PEL) [37]. Both aerobic glycolysis and fatty acid synthesis are elevated in PEL cells, which express the upregulated fatty acid synthesizing enzyme FASN and are more sensitive to the FASN inhibitor [37,38]. Interestingly, short-chain fatty acids (SCFAs) from periodontal pathogens inhibit Class 1 and 2 histone deacetylases (HDACs) and histone-lysine *N*-methyltransferases (HLMTs) to activate KSHV replication and infection by inducing active histone acetylation and suppressing repressive histone methylation [39].

## 4. S-Adenosylmethionine (SAM)

S-Adenosylmethionine (SAM) is a major methyl donor that transfers various methyl groups to DNA or histone residues by DNA methyltransferase (DNMT) or histone methyltransferases (HMT), respectively. The end product of SAM-involved methylation is S-adenosyl homocysteine (SAH) that can competitively interact with DNMTs or HMTs and inhibit their activities [40]. Therefore, modulation of SAM levels or the enzymic activities of DNMTs and HMTs could affect herpesvirus infection by targeting cellular or viral factors.

### 4.1. SAM and DNA Methylation

**Figure 1:**
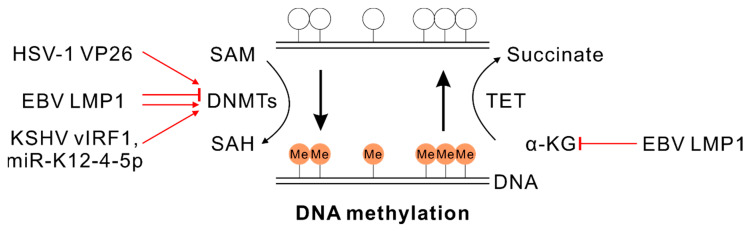
Herpesviruses regulate DNA methylation by targeting metabolic enzymes or intermediates. EBV-encoded LMP1 can promote or inhibit DNMT activity dependent on the specific cell types. Herpesviruses-associated regulation is highlighted with a red line. DNA methylation is labeled with an orange solid circle. DNMT, DNA methyltransferase; SAM, S-adenosylmethionine; SAH, S-adenosyl homocysteine; TET, ten-eleven translocation methylcytosine dioxygenase; α-KG, α-ketoglutarate.

The DNA methyltransferase (DNMT) family comprises three classical DNMT enzymes (DNMT1, DNMT3A and DNMT3B). They utilize a conserved mechanism by which a methyl group is transferred to DNA and functions in epigenetic regulation [41]. The critical roles of DNMTs in herpesvirus infection are broadly studied, and their transcription-suppressive activities are largely focused on (Figure 1).

HSV-1 capsid protein VP26 is associated with DNMT3A, and this interaction may have a potential role in HSV-1 infection [42]. Additionally, HHV-6B induces genomic hypomethylation that facilitates gene expression and viral integration during acute infection. However, the relationship between these epigenetic modifications and metabolic regulation needs further investigation [43].

EBV infection of germinal center (GC) B-cells leads to upregulation of DNMT3A but interestingly downregulates both DNMT1 and DNMT3B expression [44]. Further experimentation showed that LMP1 was responsible for the downregulation of DNMT1, while viral latent promoter Wp was bound by DNMT3A for methylated modifications at this regulatory region [44]. The heavily methylated Wp promoter was also validated in GC B-cell-derived LCL cells as well as two other cell lines (Rael Burkitt’s lymphoma cells and 11W LCL cells) [44]. Furthermore, in EBV latently infected B-cells, the important cellular proapoptotic Bim protein was inhibited by EBV latent antigens (EBNA3A and EBNA3C) to promote cell survival, which was associated with DNMT- and HDAC-mediated repressive epigenetic modifications [45,46]. However, stable knockdown of DNMT1 or DNMT3B did not affect the restricted latency of EBV-infected B-cells [47]. Additionally, in NPC cells, EBV LMP1 activates DNMT1 through the JNK-AP-1 pathway and can also recruit DNMT1 and histone deacetylases, resulting in hypermethylation and suppression of E-cadherin [48].

KSHV interacts and recruits DNMT3A to increase de novo promoter methylation of H-cadherin and inhibit its expression [49]. KSHV miR-K12-4-5p represses retinoblastoma (Rb)-like protein 2 (Rbl2) protein level and increases DNA methyltransferase-1, -3A and -3B expression, which modulates the global DNA methylation status in KSHV-positive cells [50]. KSHV-encoded viral interferon regulatory factor 1 (vIRF1) enhances DNMT1 expression by inhibiting p53 function, which further affects the methylation levels of downstream targets [51].

### 4.2. SAM and Histone Methylation

**Figure 2:**
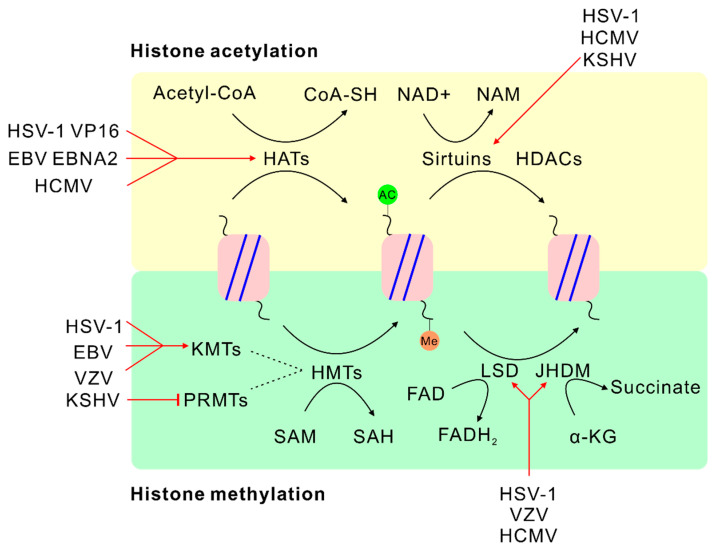
Herpesviruses modulate histone methylation and acetylation through metabolic reprogramming. Herpesviruses-associated regulation is highlighted with a red line. Histone methylation and histone acetylation are labeled with orange and green solid circles, respectively. Acetyl-CoA, acetyl-coenzyme A; CoA-SH, Coenzyme A; NAD^+^, nicotinamide adenine dinucleotide; NAM, nicotinamide; HAT, histone acetyltransferase; HDAC, histone deacetylase; HMT, histone methyltransferase; KMT, lysine methyltransferase; PRMT, arginine methyltransferase; LSD, lysine-specific demethylase; JHDM, JmjC-family histone demethylase; FAD, flavin adenine dinucleotide; FADH2, flavin adenine dinucleotide in hydroquinone form; α-KG, α-ketoglutarate.

Histone methyltransferases (HMTs) are divided into lysine methyltransferases (KMTs) and arginine methyltransferases (PRMTs), both of which can transfer methyl groups from SAM to lysine and arginine residues of histones [52,53]. Each group comprises several different members. For example, KMTs include SUV39H1, G9a, GLP, EZH2 and SETD2; PRMTs include nine members from PRMT1 to PRMT9 [54,55,56]. HMTs can transfer up to three methyl groups to lysine residue, forming mono-, di- and trimethylated derivatives, or to arginine residue, generating mono- or di- (asymmetric or symmetric) methylated derivatives [52,54]. They have been determined to regulate epigenetics and modulate herpesvirus infection (Figure 2).

EBV infection induced the expression of enhancer of zeste homolog 2 (EZH2), a major histone H3K27 methyltransferase [57]. EZH2 knockout decreased H3K27 modifications and restricted cell growth of EBV latently infected cells, while it increased viral gene expression and progeny production during lytic replication [57]. These findings suggest that EZH2 protein may be a restricted factor for the maintenance of EBV latency. Moreover, the inhibitors of EZH2 and EZH1 (EZH2/1) suppressed repressive H3K27me3 modifications. Unexpectedly, they blocked viral lytic replication through induction of cellular antiviral response, which was also efficient in inhibiting HSV and HCMV infection [58]. Knockdown of Set1, another H3K4 methyltransferase, specifically decreased H3K4me3 levels on the HSV-1 genome and inhibited viral transcription and replication during HSV-1 infection [59]. In VZV lytic infection, host cell factor-1 (HCF-1) was necessary to recruit the histone methyltransferases Set1 and mixed-lineage leukemia 1 (MLL1), further resulting in activated histone H3K4 trimethylation and induced viral IE expression [60].

KSHV-encoded ORF59 protein interacts with a protein arginine methyltransferase 5 (PRMT5) and disrupts PRMT5-induced H4R3me2s modification, which leads to the formation of active chromatin regions for gene expression in lytic reactivation [61].

## 5. α-Ketoglutarate (α-KG) and Flavin Adenine Dinucleotide (FAD)

Both α-KG and FAD are important metabolites of the TCA cycle and function in multiple metabolic processes. α-KG is produced from glucose-derived isocitrate in the mitochondria by isocitrate dehydrogenase (IDH) or synthesized from several amino acids [62,63]. Mutations in IDH1 and IDH2 can convert α-ketoglutarate to 2-hydroxyglutarate (2-HG), which is structurally similar to α-ketoglutarate and promotes H3K9 methylation via inhibition of JHDM3C/KDM4C-mediated histone demethylation [64,65,66]. FAD is a redox coenzyme that is produced from riboflavin and is involved in many metabolic reactions [67].

### 5.1. α-KG and DNA Demethylation

α-KG and Fe^2+^ can function as cofactors and substrates in ten-eleven translocation (TET) methylcytosine dioxygenase induced DNA demethylation [68]. TET enzymes convert 5-methylcytosine (5mC) to 5-hydroxymethylcytosine (hmC) by utilizing α-KG in a Fe^2+^-dependent demethylation pathway [69,70,71]. It is no surprise that herpesviruses modulate the production of α-KG to control DNA methylation levels of viral or cellular genes (Figure 1). EBV latent membrane protein LMP1 promotes the accumulation of fumarate and reduction of α-KG. These metabolic changes inactivate TETs, which then mediate the hypermethylation of the RIP3 promoter and inhibit RIP3 expression in NPC cells. This allows EBV-infected cells to escape from RIP3-dependent necroptosis [72].

### 5.2. α-KG or FAD and Histone Demethylation

Two conserved classes of histone demethylases, lysine-specific demethylase (LSD) and JmjC-family histone demethylase (JHDM), are responsible for histone demethylation [73,74,75]. Lysine-specific demethylase 1 (LSD1, also known as KDM1A), can promote H3K4 and H3K9 demethylation, while LSD2 specifically targets H3K4 demethylation [76,77,78]. Both LSD1 and LSD2 require FAD as a cofactor [79]. In contrast, JHMD catalyzes specific mono-, di- and trimethylated lysine residues for histone demethylation, but JHMD uses α-KG and Fe^2+^ for enzyme activity [73,80]. JHDM1 was found to specifically demethylate H3K36 [74]. JMJD2A is specific for the demethylation of H3K9/K36me3, and JMJD3 is specific to remove the methylation of H3K27me3 [81,82]. Herpesviruses can control histone demethylation through regulating the activities of these histone demethylases or the expression of related cofactors (Figure 2).

Both herpes simplex virus (HSV) and varicella zoster virus (VZV) infection can increase repressive H3K9 methylation levels. However, the viruses hijack the host cell factor-1 (HCF-1) to recruit histone demethylase LSD1 for expression of viral immediate-early genes through inhibition of H3K9 methylation [83]. Two demethylases, JHDM2A and LSD1, are responsible for the demethylation of mono- or dimethylated H3K9 based on the distinct cell-type specificities [84]. The involvement of HCF-1 also increases H3K4 trimethylation in coordination with Set1 and MLL1 [60,85]. The initial infection of HSV-1 and HCMV requires LSD1 and Jumonji C domain-containing protein 2 (JMJD2), two histone demethylases that are responsible for activation of viral immediate-early gene expression [86]. Furthermore, inhibition of LSD1 activity with the monoamine oxidase inhibitor tranylcypromine (TCP) reduces viral lytic infection and reactivation in vivo, suggesting the critical roles of histone demethylation during HSV-1 infection [87]. Additionally, JMJD3 but not JMJD2A can induce KSHV reactivation, suggesting the important roles of H3K27me3 in the maintenance of KSHV latency [88].

## 6. Acetyl-Coenzyme A (Acetyl-CoA)

Histone acetylation, which leads to an open chromatin architecture and active transcriptional regulation, is one of the best-characterized epigenetic modifications [89]. Acetyl-CoA is a critical metabolic intermediate for cellular catabolism and anabolism and acts as an essential substrate of histone acetyltransferases (HATs) [62,90]. The expression level of acetyl-CoA can affect HAT-mediated histone acetylation. Thus, acetyl-CoA controls various important cellular functions by restricting epigenetic modifications of gene expression [91], which has also been studied in herpesvirus infection (Figure 2).

Histone acetylation activates EBV lytic replication by inducing expression of immediate-early genes (*BZLF1* and *BRLF1*), while histone deacetylation is important for the maintenance of EBV latency [92,93]. EBV-encoded EBNA2 and HSV-encoded VP16 activate transcription through utilization of the histone acetyltransferases activities of p300, CBP and PCAF [94,95]. Furthermore, EBNA2-mediated expression of viral Cp and LMPp promoters is associated with promoter-specific H3 or H4 acetylation [96]. Epigallocatechin-3-gallate (EGCG), a histone acetyltransferase inhibitor, specifically targets the majority of HAT enzymes and recruits HDAC3 but abolishes p300 binding to modulate the *interleukin-6* promoter, which may inhibit EBV-induced transformation [97]. This suggests the critical role of HAT or HDAC modifications during EBV-mediated oncogenesis.

Nevertheless, metabolic regulation of various intermediates has strong interaction. For example, acetyl-CoA carboxylase (ACC1) is a critical enzyme in fatty acid synthesis as it catalyzes carboxylation of acetyl-CoA to malonyl-CoA [98]. HCMV induces the expression of ACC1 mRNA and protein and enhances its enzymatic activity, while ACC1 inhibition attenuates HCMV replication that suggests an important role in viral infection [99]. Interestingly, acetyl-CoA is also an essential metabolite for supplying histone acetylation, and increased ACC1 expression may enhance de novo fatty acid synthesis by consuming acetyl-CoA. This leads to suppression of histone acetylation in the nucleus [100]. 

## 7. Nicotinamide Adenine Dinucleotide (NAD^+^)

NAD^+^ is a cofactor of histone deacetylation that is mediated by histone deacetylases known as sirtuins (SIRTs) [101,102,103]. NAD-dependent deacetylase sirtuins are class III histone deacetylases (HDACs) that are evolutionarily conserved and dynamically regulate the cellular metabolic state. This suggests a tight relationship between metabolism and epigenetics in regulation of gene expression [104]. A small interfering RNA (siRNA) screen targeting each sirtuin showed that HCMV and HSV-1 production increased after knockdown of sirtuins, demonstrating the antiviral activity of sirtuins during infection [105]. Further experiments with sirtuin-modulating drugs verified that HCMV production could be induced by a SIRT1 inhibitor that specifically inhibits its deacetylation activity [105,106]. Additionally, treatment with SIRTs inhibitors, nicotinamide (NAM) or sirtinol, induced expression of viral lytic genes (RTA/ORF50, ORF57, ORF59 and ORF65) in KSHV latently infected cells [107]. Similar to NAM treatment, the absence of SIRT1 was sufficient to increase the active H3K4me3 mark and the repressive H3K27me3 mark at the RTA promoter, which promoted KSHV lytic reactivation [107]. SIRT1 regulation is also dependent on the cellular NAD^+^ concentrations. Thus, these findings suggest a direct relationship between metabolic regulation and cellular epigenetics during the KSHV life cycle (Figure 2).

Other HDACs do not require NAD^+^ as a cofactor, but their functions are also related to metabolic regulation and play important roles during herpesviruses infection. HSV-1 infected cell protein 0 (ICP0) specifically interacts with class II HDACs 4, 5 and 7 and suppresses their activities, but it does not reduce the global deacylated activities [108]. ICP0 inhibits the repressive REST/CoREST/HDAC activity and enhances viral gene expression through displacement of HDAC1 or HDAC2 from this histone deacetylase complex [109,110,111]. Moreover, ICP0 promotes histone acetylation of viral genes in HSV-1 lytic infection or HSV-2 superinfection [112,113]. Two histone deacetylase inhibitors, sodium butyrate and trichostatin A (TSA), can also reactive HSV-1 latency in neuronal cells [114].

Phase 1 and 2 clinical trials of the pan-histone deacetylase (pan-HDAC) inhibitor arginine butyrate in combination with the antiviral drug ganciclovir showed significant potential for treatment of EBV-induced lymphoid malignancies [115]. Moreover, another three HDAC inhibitors (LBH589 (panobinostat), MS275 (entinostat) and largazole) were determined to effectively sensitize EBV-associated lymphomas to ganciclovir [116]. The presence of HDAC3 (class I HDAC) in nonpermissive cells inhibited the activity of HCMV major immediate-early promoter (MIEP), while HDAC inhibition induced viral transcription through enhancing histone acetylation of MIEP [117].

## 8. Glutaminolysis

Glutamine is a nonessential amino acid but is rapidly consumed for proliferation and survival in cancer cells [118,119]. As an important metabolic precursor, glutamine is converted to glutamate by glutaminase in glutaminolysis, which is further oxidized into α-ketoglutarate, a necessary intermediate in the TCA cycle [119,120]. Herpesviruses require glutaminolysis to support cellular energy during infection.

HCMV-infected cells require more ATP production, which is provided through conversion of glutamine for entry into the tricarboxylic acid (TCA) cycle. This also requires the enhanced activities of glutaminase and glutamate dehydrogenase [121]. Lack of glutamine after HCMV infection leads to inhibition of viral production, which demonstrates that glutamine metabolism is necessary for HCMV infection [121]. 

KSHV induces the increase of intracellular glutamine levels and the uptake of glutamine, which prevents apoptotic cell death in KHSV-infected endothelial cells [122]. The tricarboxylic acid (TCA) cycle can utilize the intermediates α-KG and pyruvate to support cell survival in the latently infected cells when glutamine is absent [122]. KSHV can also modulate the Myc/MondoA complex to activate SLC1A5 expression, a glutamine transporter that enhances glutaminolysis for cell survival [122].

## 9. Conclusions

This review focuses on the crosstalk between epigenetics and metabolism during herpesvirus infection and demonstrates the enormous complexity of this topic. Although many studies have demonstrated epigenetic or metabolic regulation during herpesviruses infection, their interactions have not been fully explored. This may provide an opportunity to examine in greater detail how herpesviruses can establish and maintain latency. Understanding the associated mechanisms will provide information that promises the development of novel therapeutic intervention strategies for treatment of herpesviruses-related human diseases.
